# Use of an Insulin Pump in the Elderly Surgical Patient: Tolerance of Total Pancreatectomy After Neoadjuvant Chemotherapy for Multifocal Pancreatic Cancer

**DOI:** 10.1089/pancan.2018.0017

**Published:** 2018-10-25

**Authors:** Andrew McGregor, Daniel Kleiner

**Affiliations:** Department of Surgery, Danbury Hospital, Danbury, Connecticut.

**Keywords:** diabetes, insulin pump, pancreatic cancer

## Abstract

**Introduction:** Pancreatic cancer is one of the most fatal cancers if not caught early and is associated with late disease presentation. Multifocal pancreatic cancer is particularly difficult to treat as cases that are amenable to surgical resection require total pancreatectomy. Such patients will develop brittle diabetes as they require exogenous insulin after surgery and in the apancreatic state lose counter-regulatory homeostatic mechanisms (i.e., glucagon). We present an elderly patient who underwent neoadjuvant chemotherapy and total pancreatectomy. The patient has adequate glycemic control postoperatively being managed with an insulin pump and remains disease free at 3 years and 3 months after resection.

**Case Presentation:** A 72-year-old male presented with two tumors, in the head and tail of the pancreas, respectively, which were consistent with pancreatic adenocarcinoma by endoscopic ultrasound biopsy. Neoadjuvant FOLFIRINOX had been administered and total pancreatectomy was performed. The patient did well postoperatively and was discharged on postoperative day 8. The patient was seen by endocrinology pre- and postoperatively who started an insulin pump for glycemic management 2 weeks postoperatively. The patient's HbA1c was 7.9% at 3 months. The patient remains disease free at 3 years and 3 months with an HbA1c of 7.0% and a normal CA19-9.

**Conclusion:** This case highlights that glycemic control after total pancreatectomy with the use of an insulin pump in the elderly population is achievable. Elderly patients can struggle with certain technologies and selecting appropriate patients for insulin pump therapy after total pancreatectomy is imperative.

## Introduction

Poor glycemic control after pancreatic surgery is a well-known consequence. Total pancreatectomy renders the patient completely dependent on the use of exogenous insulin. Known as “brittle diabetes,” systemic complications of diabetes after pancreatic surgery are problematic as blood sugar control can be difficult in the elderly population. Traditional methods of insulin delivery include subcutaneous injections and insulin pumps. Newer technology has allowed the use of continuous glucose monitoring to achieve better hemoglobin A1C levels. We present a patient who underwent neoadjuvant chemotherapy and then total pancreatectomy for multifocal pancreatic adenocarcinoma. He was bridged to an insulin pump 2 weeks postoperatively and has been able to maintain adequate blood glucose control without any complications of his diabetes postoperatively.

## Case Presentation

A 72-year-old retired male engineer was diagnosed with two pancreatic lesions, one measuring 2 cm at the head of the pancreas and the other measuring 1.5 cm at the tail of the pancreas. Endoscopic ultrasonography with biopsies confirmed pancreatic adenocarcinoma in both lesions.

**FIG. 1.**
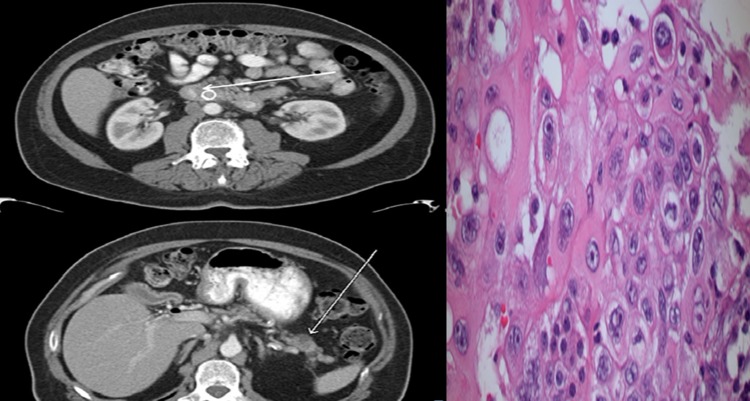
Left: axial imaging demonstrating head and tail pancreatic tumors. Right: H + E staining demonstrating pancreatic adenocarcinoma (magnification = 400 × ).

The patient underwent seven cycles of neoadjuvant chemotherapy with FOLFIRINOX followed by total pancreatectomy (Fig. 1). The specimen pathology revealed two foci of moderately differentiated pancreatic adenocarcinoma with perineural invasion, but no lymphovascular invasions. Largest dimension was 1.4 cm in the tail of the pancreas and 1.3 cm in the head of the pancreas. Margins were negative and 25 lymph nodes were benign. The patient was initially started with exogenous pancreatic enzyme supplements and long-acting insulin in the hospital and was transitioned to an insulin pump postoperatively. The patient was followed by endocrinology pre- and postoperatively where insulin pump education was given before pancreatectomy. The patient's preoperative HbA1c was 5.9%, 3-month postoperative HbA1c was 7.9%., and 3-year postoperative HbA1c was 7.0%. He remains disease free at 3 years and 3 months with a normal CA-19-9. The patient has been able to easily manage his insulin pump to control his blood glucose levels.

## Discussion

Total pancreatectomy historically has been a morbid procedure for patients. Total pancreatectomy renders patients completely dependent on exogenous insulin and pancreatic enzymes. The role of total pancreatectomy is limited in benign and malignant pancreatic pathology. However, Billings et al. did show that even though quality of life is slightly decreased after total pancreatectomy, the differences were subtle and concluded that total pancreatectomy is a viable option when needed.^1^

One long-term consequence after total pancreatectomy is the development of “brittle diabetes.” Glycemic control can be difficult after pancreatectomy as patients are reliant on insulin. Jethwa et al. showed that patients who underwent total pancreatectomy had a median HbA1c level of 8.2%, which was not significantly different from the matched type 1 diabetic control group. They also demonstrated that glycemic control was better in patients undergoing total pancreatectomy for carcinoma versus chronic pancreatitis.^2^

Insulin pumps have been used since the 1990s as a way for diabetic patients to better manage blood sugar than the standard subcutaneous injection methods. A meta-analysis performed by Weissberg-Benchell et al. showed that patients undergoing continuous glucose injection by a pump device have better glycemic control than patients who used traditional methods of insulin injection.^3^ Further advancements in glucose control include continuous glucose monitoring systems. Deiss et al. demonstrated a reduction of HbA1c levels up to 2% in patients undergoing continuous glucose monitoring.^4^

In conclusion, glycemic control after total pancreatectomy can be achieved. Elderly patients should be considered for continuous insulin delivery systems to manage their diabetes after total pancreatectomy. The continued success of this patient's glucose control was partially dependent upon the early initiation of perioperative glucose management education. Finally, total pancreatectomy remains a viable option for resection in selected patients who are able to successfully manage their glucose levels postoperatively.
